# Assessing Team Performance: A Mixed-Methods Analysis Using Interprofessional *in situ* Simulation

**DOI:** 10.5811/westjem.18012

**Published:** 2024-06-11

**Authors:** Ashley C. Rider, Sarah R. Williams, Vivien Jones, Daniel Rebagliati, Kimberly Schertzer, Michael A. Gisondi, Stefanie S. Sebok-Syer

**Affiliations:** * Stanford University, Department of Emergency Medicine, Palo Alto, California; † Royal College of Surgeons, Dublin, Ireland; ‡ Albany Medical College, Albany, New York

## Abstract

**Introduction:**

Optimizing the performance of emergency department (ED) teams impacts patient care, but the utility of current, team-based performance assessment tools to comprehensively measure this impact is underexplored. In this study we aimed to 1) evaluate ED team performance using current team-based assessment tools during an interprofessional in situ simulation and 2) identify characteristics of effective ED teams.

**Methods:**

This mixed-methods study employed case study methodology based on a constructivist paradigm. Sixty-three eligible nurses, technicians, pharmacists, and postgraduate year 2–4 emergency medicine residents at a tertiary academic ED participated in a 10-minute in situ simulation of a critically ill patient. Participants self-rated performance using the *Team Performance Observation Tool* (TPOT) 2.0 and completed a brief demographic form. Two raters independently reviewed simulation videos and rated performance using the TPOT 2.0, *Team Emergency Assessment Measure* (TEAM), and *Ottawa Crisis Resource Management Global Rating Scale* (Ottawa GRS). Following simulations, we conducted semi-structured interviews and focus groups with in situ participants. Transcripts were analyzed using thematic analysis.

**Results:**

Eighteen team-based simulations took place between January–April 2021. Raters’ scores were on the upper end of the tools for the TPOT 2.0 (R1 4.90, SD 0.17; R2 4.53, SD 0.27, IRR [inter-rater reliability] 0.47), TEAM (R1 3.89, SD 0.19; R2 3.58, SD 0.39, IRR 0.73), and Ottawa GRS (R1 6.6, SD 0.56; R2 6.2, SD 0.54, IRR 0.68). We identified six themes from our interview data: team member entrustment; interdependent energy; leadership tone; optimal communication; strategic staffing; and simulation empowering team performance.

**Conclusion:**

Current team performance assessment tools insufficiently discriminate among high performing teams in the ED. Emergency department-specific assessments that capture features of entrustability, interdependent energy, and leadership tone may offer a more comprehensive way to assess an individual’s contribution to a team’s performance.

Population Health Research Capsule What do we already know about this issue?

*Emergency department teams are dynamic and complex, with both individual and team factors that impact patient care.*

What was the research question?

*We aimed to understand the ability of performance tools to assess ED teams as well as identify characteristics of effective teams.*

What was the major finding of the study?

*ED teams in the simulation were rated highly on all tools with good interrater correlations 0.46, 0.68, and 0.72 for each of the tools.*

How does this improve population health?

*A better understanding of interdependent team factors will allow us to educate and train more effective patient care teams.*

## INTRODUCTION

Patient care in the emergency department (ED) depends on highly effective interprofessional teams. ED teams are dynamic, complex to train, and subject to the preparedness of individual team members while caring for critically ill patients. Although team training has been championed by the National Academy of Medicine to reduce adverse events, the fluid nature of ED teams makes such training complex.^1^ Additionally, individual team member contributions can influence the readiness of an ED team. Previous research has shown that individual performance and communication failures are substantial contributors to adverse events,^2^^,^^3^ affecting the interdependent nature of team-based care.^4^^,^^5^ Therefore, evaluating how well existing team performance assessments are at capturing individual and team-based performance is necessary to ensure accurate measurement of teams under the direst circumstances.

The Agency for Healthcare Research and Quality and the US Department of Defense rigorously developed measures that evaluate teamwork.^1^ The most widely used tool for assessing team performance and patient safety is the Team Strategies and Tools to Enhance Performance and Patient Safety (TeamSTEPPS) *Team Performance Observation Tool* (TPOT), now with a second version, TPOT 2.0. This 23-item instrument integrates five areas of competence: team leadership; team structure; situation monitoring; mutual support; and communication.^6^ The *Team Emergency Assessment Measure* (TEAM) is an alternate 12-item tool that also measures team performance, but it was designed specifically for team assessment in the ED setting.^7^ The *Ottawa Crisis Resource Management Global Rating Scale* (Ottawa GRS) is another tool that assesses crisis resource management skills of leader-team member interactions.^8^ Although these tools have some validity evidence,^8^^–^^13^ the extent to which they reliably and accurately measure team-based performance in various contexts warrants further investigation to understand how to best assess an individual’s contributions to ED team performance.

Further complicating team assessment is the critical role of dyads^14^ and the interdependence of individuals within teams.^4^^,^^15^ Interprofessional members of the ED team are inseparably tied to one another, and often there is no choice whether someone becomes part of the team. In the ED, teams are formed out of necessity to provide acute care for critically ill patients. These circumstances essentially require immediate entrustment among individual team members, which is not always feasible or realistic. Underlying the theory of interdependence is the idea that some pairings of team members will be more effective than others; therefore, identifying key factors that influence team member dynamics is critical.^14^^,^^15^ This conceptual framing has implications for how ED team-based performance (ie, where teams rapidly form to meet the emergent needs of patients) is assessed.

Due to our incomplete understanding of the important elements that contribute to individual and team performance, we set out to explore the effectiveness of current team-based performance assessment tools in the ED setting. Using an interprofessional, in situ simulation, we aimed to do the following in this study: 1) evaluate the effectiveness of TPOT 2.0, TEAM, and Ottawa GRS team-based assessment tools in the ED setting; and 2) identify characteristics of effective teams that are attributable to individuals and may not be captured within existing team-based assessments.

## METHODS

We used mixed-methods case study methodology in the context of a team-based, in situ simulation to explore the effectiveness of team-based assessments and explore the relationship between team dynamics and individuals’ team-based performance.^16^^,^^17^ We used a constructivist paradigm, which holds that an individual’s perspective is the basis for reality and that multiple, socially constructed realities can exist at once for this research.^18^^,^^19^ We chose case study methodology to understand the various perspectives of team participants and observers in the context of an ED-based simulation.

All ED nurses, technicians, pharmacists, and postgraduate year (PGY) 2–4 emergency medicine residents within one academic health system were eligible to participate in this study. We excluded PGY-1 residents due to their limited experience leading resuscitations. The study took place in a large, suburban, academic ED at a tertiary care facility. We conducted simulations twice per week during low-volume hours; strict policies for cancellation were followed based on ED volume and patient care needs. The Stanford University Institutional Review Board approved this study (#55327).

### Quantitative Study Design and Data Collection

Using convenience sampling, we solicited volunteers to participate in simulations held over a four-month period (January–April 2021). Each simulation included a nurse, a resident, a pharmacist and, in some cases, an ED technician. Attendings were not included to ensure that patient care would not be disrupted. Prior to the simulated case, the 63 participants received a two-minute pre-brief from an in-person facilitator on expectations for the simulation, goals of the session, and confidentiality. We obtained written consent for study participation and video recording of the simulations, and participants could opt out of the study at any time.

We conducted a simulated case of a patient presenting with sepsis and an arrhythmia using the high-fidelity HAL patient simulator (Gaumard Scientific Company Inc, Miami, FL) and equipment props that are typically available in an ED patient room. The ED pharmacist supplied simulated critical care medications for use during the scenario. We recorded the simulation for asynchronous rating. The case was followed by a 10–15 minute debrief with all team members, which was not recorded to protect the psychological safety of participants. After the debrief, the participants completed a self-rating for the entire team using the TeamSTEPPS TPOT 2.0 to increase familiarity with the components of TeamSTEPPS, as well as a brief demographic form that included training year/years of work experience, age, and gender. We omitted items 2d, 5c, and 5d on the TPOT 2.0, as these were not relevant to our study protocol.

We recruited two board-certified emergency physicians from outside institutions to assess the simulation video recordings. The two raters underwent a two-hour training session where they were introduced to the project and the three instruments. The facilitator also reviewed an example case, which the reviewers independently scored and were then calibrated against each other. The raters subsequently watched an example video and deliberated each item on the scoring sheet until they arrived at a consensus. Raters then independently reviewed all recorded simulations for which consent was provided by all team members. Raters assessed team performance with the TPOT 2.0 and TEAM. They assessed leadership by completing the leadership categories on TPOT and TEAM, as well as the Ottawa GRS. Only the TPOT 2.0 assessment by the raters was used for comparative data analysis to use objective third-party ratings rather than the self-assessment from participants.

### Qualitative Study Design and Data Collection

We invited all volunteer staff participants via email who completed the simulation component of the study to participate in an individual, semi-structured interview via Zoom (Zoom Video Communications, San Jose, CA). A total of 10 ED staff members volunteered to participate in the semi-structured interview, including five nurses, four pharmacists, and one ED technician, and each participant received a $25 gift card as compensation for their time. We also conducted two focus groups with five resident team leaders. Each session lasted 30–60 minutes. A single female interviewer (AR) conducted all interviews and focus groups with predetermined questions that were then allowed to progress to open dialogue.

### Data Analysis - Quantitative

We collected demographic information and calculated measures of central tendency for each group. We also analyzed rater’s average scores and standard deviations for each of the tools. We performed a correlation analysis of the within-rater and between-rater scores on each tool. We also compared team-based leader performance based on the Ottawa GRS with the leadership subset on the TEAM and TPOT 2.0. We generated validity evidence^20^^,^^21^ for the TPOT 2.0 using content validity, internal structure, and relationship to other variables. Content validity was assessed by examining which performance measures participants thought should be included in an assessment tool. We examined internal structure by assessing correlations between the inter-rater reliability and self vs rater scores. Relationship to other variables was manifested as concurrent validity by comparing the tools. We performed data analysis using IBM SPSS v 27 (SPSS Inc, Chicago, IL) and Microsoft Excel v 16.6 (Microsoft Corporation, Redmond, WA).

### Data Analysis - Qualitative

Of the 63 participants, 15 (24%) agreed to the interview. We transcribed and anonymized the interviews using the HIPAA-compliant software TranscribeMe! (TranscribeMe Inc, Oakland, CA) program. Two coders (VJ and DR) who were not involved in either the simulation or interview process underwent qualitative training consisting of pre-reading on thematic analysis and completion of a Dedoose webinar v 9.0.17 (Dedoose, Manhattan Beach, CA) webinar. Coders completed a one-hour training session using an excerpt of a transcript to demonstrate the coding process. A second excerpt was done in real time. The coders were then given five days to code the first transcript. This was reviewed by both coders and other members of the research team to discuss and identify patterns. Coders then read all transcripts prior to starting the first coding round. In accordance with Braun and Clarke’s six phases of analysis,^22^ after complete read-through of the coded transcripts, coders then generated initial codes on the second review.

After the initial round, two researchers (VJ and DR) discussed and refined all independently created codes. Consensus was achieved with review of each transcript on a unified code list. Two other members of the research team (AR and SW) then reviewed the transcripts and codes to develop themes. Investigator triangulation of themes, with attention to the quantitative findings, was performed by a third member of the research team (SS). Initial code and excerpt to theme categorization resulted in 67% independent agreement between the two secondary reviewers. Coding was then revised, consolidated, and modified based on consensus. Two researchers (AR and SW) performed a round of focused re-coding and theme generation, and a final reviewer (AR) performed the last round of code review and edits within existing themes.

Regarding reflexivity, both coders (DR and VJ) had significant experience with healthcare teams and crisis resource management as prior simulation technicians, but were not employed full-time in the ED. While this limited their context for some of the qualitative analysis, it allowed them to focus on teamwork and leadership features without preconceived notions. The code reviewers (AR and SW) are emergency physicians who practice at the academic health center where the study was conducted. Both code reviewers have been involved in residency program leadership. AR facilitated all the interviews but was blinded to the identity of residents and staff during coding.

## RESULTS

### Quantitative Analysis

We completed 18 simulations with 63 participants from January–April 2021. Some cases had a pharmacist who had participated in multiple simulations (due to the limited number of clinical pharmacists employed in the ED); otherwise, participants were part of a scenario only once. Participant demographics are listed in Table 1 along with the mean self-rated TPOT 2.0 score. Missing data points were omitted from the analysis.

**Table 1.** Demographic characteristics and mean score on Team Performance Observation Tool 2.0.

Group	Years of experience	Male	Female	Mean score on self-rated TPOT
Residents	PGY-2 (7 residents) PGY-3 (5 residents) PGY-4 (6 residents)	14	4	84
Nurses	8 (3–30)	5	11	93
Techs	3 (1–10)	5	7	90
Pharmacists	5 (1–17)	11	6	88

*PGY*, postgraduate year; *TPOT*, Team Performance Observation Tool.

The descriptive statistics of rater scores on each scenario were on the upper end of the scale for each of the tools. The two raters’ scores clustered high for the five-point TPOT 2.0 (R1 4.90, SD 0.17; R2 4.53, SD 0.27), the four-point TEAM tool (R1 3.89, SD 0.19; R2 3.58, SD 0.39), and the seven-point Ottawa GRS tool (R1 6.6, SD 0.56; R2 6.2, SD 0.54). All three scales were noted to have scores that crowded around the maximum. There were high correlations of total score for a given case reviewed within the same rater, particularly for TEAM and Ottawa GRS. Inter-rater correlations were 0.46, 0.68, and 0.72, respectively, for the TPOT 2.0, Ottawa, and TEAM (Table 2). Year in residency (PGY-2, PGY-3, PGY-4) was not correlated to raters’ scores on each of the tools.

**Table 2.** Inter-rater correlations for each team and leader performance tool.

Rater and tool	Rater 1 TPOT	Rater 2 TPOT	Rater 1 Ottawa	Rater 2 Ottawa	Rater 1 TEAM	Rater 2 TEAM
Rater 1 TPOT	1.00					
Rater 2 TPOT	0.46	1.00				
Rater 1 Ottawa	0.89	0.35	1.00			
Rater 2 Ottawa	0.44	0.52	0.68	1.00		
Rater 1 TEAM	0.71	0.27	0.92	0.69	1.00	
Rater 2 TEAM	0.45	0.54	0.66	0.94	0.73	1.00

*TPOT*, Team Performance Observation Tool; *Ottawa*, Ottawa Crisis Resource Management Global Rating Scale;

*TEAM*, Team Emergency Assessment Measure.

### Qualitative Analysis

We identified six themes related to the individual and team-based performance (Table 3), including the following: 1) *team member entrustment*; 2) *interdependent energy*; 3) *leadership tone*; 4) *optimal communication*; 5) *strategic staffing*; and 6) *simulation empowering team performance*.

**Table 3.** Themes reflecting effective leadership and team performance.

Theme	Description	Exemplary quotes
Team member entrustment	The expectation of team members to competently execute their interprofessional tasks without supervision or interjection and have a substantial cross-understanding of roles to provide support of other team member tasks through anticipation and automaticity.	*•I guess having trust, also, that, for example, we need IV access. I need to give epinephrine or whatever. Just having that trust that your team members are going to be able to carry that out, and that you don’t have to worry about, “Okay. Is this happening? Is this not happening?” So having that interpersonal trust between you, your provider, your other teammates is really important. RN, participant E* •*People are that well trained and things happen automatically, right? You don’t need the doctor to be like, “Hey, can we get an IV line? Can you put them on the monitor?” It happens automatically. So in that sense, I think there is a very good understanding, at least in my situation, of where everyone falls into place. Pharmacist, participant H*
Interdependent energy	The ability for one individual to influence others with non-verbal cues and general disposition that in turn impacts the energy and performance of team members.	•*So if they’re, I guess, I don’t want to say outgoing, but if they’re soft spoken, it tends to be a little bit more of a struggle. And then I think that if they are-- yeah. I think generally, if they’re a warmer person, the team tends to rally around with a little bit more excitement or a little bit more energy versus someone with a more flat affect, then everyone comes in kind of to match that. Pharmacist, participant J* •*That is a skill, for you to kind of see someone going through a very critical situation, to be able to transform the energy into something positive. RN, participant C*
Leadership tone	The ideal demeanor of a leader that balances collaborative and decisive actions while maintaining continuous open communication and vulnerability with the team.	•*I think having a demeanor that’s sort of open and makes people comfortable to speak up, whether it’s with an idea they have or something they see that someone else is not doing right or anything, just feeling comfortable speaking up.* *ED tech, participant B* •*I don’t know if saying a sense of humility is the right way of saying this for the team leader but realizing that you may not know everything in every single moment.* *Resident, participant K*
Optimal communication	Communication that is individualized and spoken in an appropriate tone at an appropriate time to contribute to the shared mental model.	•*You’re saying the same words. It’s just your tone is all that’s different. It takes the same amount of time. You’re not saving any time, but your tone is imparting a sense of urgency for whatever reason. And I think that breaks down teamwork when people are having tone issues. ED tech, participant B* •*Back to communication for me, so making sure -- I don’t know how I would rate it or how I would word it, but whether there was clear instruction and clear feedback, I guess, so that way, you can determine how well something was understood or communicated between people. RN, participant E*
Strategic staffing	Team sizes should be designed to meet the needs of the patient care scenario, with smaller teams helping to optimize noise and space.	•*I think that really depends on the resuscitation you are doing. So for the scenario in our simulation in particular, I think the size of the team was perfect. You usually only need one physician and maybe a nurse, and then plus or minus pharmacy just depending on how your institution runs. But if you are running a complex traumatic resuscitation, then you’re going to need more hands, especially with CPR. Resident, participant L* •*Oh, definitely having a smaller team with more specific defined role, definitely in the aspect of crowd control it made it a lot easier. Pharmacist, participant D*
Simulation empowering team performance	Simulation is perceived as a safe environment to practice skills and critically reflect during the debrief to build up team member entrustment	•*I think that all helped us learn what people’s feelings are during a scenario like that and how we can help make a difference for those people when we’re kind of taking care of sick patients, especially patients that can change their clinical status quickly, and that that particular element can help you better take care of those patients, having that team that understands everybody else’s needs and thoughts as well. Resident, participant M* •*Yeah, I actually really did enjoy that simulation. I felt I was a bit unprepared when I was coming into it. But just being able to freely work in a safe environment, that’s not really with the patient with someone’s life in the balance, I think that’s really a great opportunity for us to be able to grow and just smooth out any kinks there, get better with our skills.* *RN, participant A*

The concept of entrustment stems from the competency-based medical education literature.^23^ In the setting of team-based performances, *team member entrustment* means trusting that a team member will be able to complete a role-specific task without oversight or specific direction. Such entrustment decisions may need to be made rapidly in the setting of ad hoc ED teams and is critical for building relationships that drive team dynamics.

Within our data, characteristics such as age and gender of team members were not perceived to impact entrustment. Our participants noted that personality and previous experience with someone managing a critically ill patient was important for team member entrustment. In the following quote, one participant comments that witnessing a leader’s ability to manage critically ill patients inspired entrustment in their leadership role. “*I don’t think it’s necessarily a number of shifts. What I think it is, it’s severity of cases. So, you might have one shift with someone and just have a killer of a day with ESI [Emergency Severity Index]1s and 2s and watched this person rock it, and you’re like, “Okay, I know they’re on it.” (Participant G, RN)* As this team member describes, familiarity was an indicator used by participants to make quick entrustment decisions in the ED setting.

*Interdependent energy* was described as the influence of confidence and demeanor that an individual team member has during a performance that appeared to alter team dynamics and impact team synergy. Several participants also mentioned the importance of tone-setting for a collaborative environment and finding a balance of humility and confidence, as highlighted by this comment about *leadership tone*. *“I have never worked with that doctor before. But I can tell just by his demeanor and his tone that he knew. He was pretty confident on what was going on. So that made me relax and kind of confident as well.” (Participant F, RN)*

*Optimal communication* was also noted as a key factor. This includes appropriate timing, directed toward a specific individual, execution using a reasonable tone, and facilitation of a shared mental model. *Strategic staffing*, specifically small teams, was described by participants to optimize performance, with examples such as keeping the noise level low and allowing for direct communication to individuals. Finally, *simulation empowering team performance* reflects that the simulation was described by participants as a way to practice skills and subsequently reflect upon the experience during an interprofessional team debrief. The session allowed team members to foster relationships, provide feedback, and build entrustment.

## DISCUSSION

We used an interprofessional, in situ simulation to evaluate team performance using multiple instruments. A mixed-methods approach allowed us to gather quantitative ratings of performance and qualitatively identify features of optimal interprofessional team performance. We found the two team assessment tools, TPOT 2.0 and TEAM, poorly discriminated when teams were assessed as functioning well together. This leaves little opportunity for capturing individual contributions to team performance for subsets of individuals within the team. Our qualitative findings also suggest that these performance measures do not capture some of the dynamic interdependent team features that drive team functionality.^5^ Moving forward, finding a way to capture dynamic features of team relationship building and interdependence can comprehensively provide a more accurate assessment of team performance.

Our findings suggest that the TPOT 2.0 lacks sufficient validity evidence for use in the ED. The overall clustering of high scores may suggest either strong performers within our sample, items that are too easy, or vague anchor points that made it difficult for raters to discriminate. Alternatively, this tool may not be optimized for differentiating individual performance within high-performing teams. The inter-relater reliability IRR of the TPOT was low at 0.46 (Table 2), which may reflect limited rater agreement and, therefore, reliability of the tool. Finally, we identified several features of team performance that participants felt were not sufficiently captured in the assessment tool, mainly entrustment features related to anticipation and automaticity, leadership tone, and interdependent energy.

Additionally, our qualitative analysis provided insight on the features of team dynamics that may be important for optimizing performance. Entrustment among fellow team members occurs when individuals serving in various interprofessional roles are trusted to function within the scope of their practice. Entrustment in our qualitative analysis was largely driven by strong role competence, anticipation, and automaticity. While competence may come from training and experience, anticipation and automaticity are uniquely important for each member of a rapidly forming ad hoc team in high-stakes situations like the ED. Because every resuscitation is slightly different, automaticity and anticipation cannot be based on an algorithm but rather on pattern-recognition and creation of shared understanding, an innately interdependent process. While anticipation is reflected in the TEAM tool, neither is explicitly represented in the TPOT. Other features that are highly important to ED teams to emphasize in performance tools included interdependent energy and tone of communication.

Situating these findings in the broader literature, ED teams are interdisciplinary action teams that task multiple, highly specialized professionals with a critical situation.^24^ Fernandez et al proposed a robust model for EM teamwork taxonomy to capture the process as well as the outcome.^25^ This includes the stages of planning processes, action processes, reflection processes, and supporting mechanisms. According to this model, teams will go back and forth between *action processes* focused on goals and *transition processes* that allow for planning. Both stages are highly dependent upon interpersonal factors between team members.

Two of the action processes—“backup behavior” of managing team members’ tasks and “coordination” of the inherently interdependent order of activities—are fundamentally dependent on this described construct of team member entrustment.^25^^,^^26^ This idea resonates with the concept of collaborative interdependence^15^ in which team members come together and leverage the strengths of one another. Entrustment may be a necessary step toward establishing a team’s collaborative interdependence as its absence may lead to a breakdown in team functioning. Our study helped discern team member actions and factors that may contribute to rapid entrustability and guide these action processes, even in ad hoc teams, including demonstration of role competency, automatic fulfillment of duties, and anticipation of next actions. To improve interprofessional team performance assessment, we need more granular resuscitation-specific performance measures that capture team member entrustment,^23^ leadership tone, and interdependence.^4^^,^^23^

### Educational Implications

Our finding that postgraduate year (PGY) level did not correlate with team performance scores highlights the challenge of assessing resuscitation leadership due to the interdependent nature of team performance.^5^^,^^27^^–^^28^ In the move toward competency-based education and implementation of Entrustable Professional Activities in the workplace,^29^ this is critically important. A PGY-2 may, for instance, be falsely assessed as fully entrustable based on the resuscitation of a patient in the clinical setting, when in fact their performance was highly influenced by other experienced team members. This underscores the inherent challenges of resident assessment in the clinical setting, due to the constant interdependent workflows with other team members. We propose that future team assessment skills involve leadership tone and energy as played out in the interdependent workflow of the team. This can only be accurately assessed in the context of interprofessional teams in the workplace through collection of both observations and gathering team member experience of tone, energy, and entrustment.

## LIMITATIONS

We performed this study at a single academic institution and, thus, the findings represent the culture and characteristics of that setting. Further research is needed to assess the transferability of our findings to other contexts. The study participants were from a convenience sample, which may limit the generalizability of these results. Furthermore, the nursing staff was noted to be very experienced with a median of eight years in practice; this may have positively influenced team performance and contributed to the high scores we observed across the tools. It is also possible that filming the scenarios may have contributed to a Hawthorne effect. While all participants were offered an opportunity to participate in the qualitative interviews, only a smaller subset did, which limits the transferability of our findings as those choosing to participate may be different than those who did not. Finally, the case used a mannequin instead of a real patient, which offers a blanket of psychological safety that a real clinical scenario does not.

## CONCLUSION

This mixed-methods study identified limitations of current tools for assessing team-based performance and offers opportunities for improvement. Future tools assessing team performance should focus on capturing entrustment, leadership tone, and interdependence.
